# Updated Prevalences of Asthma, Allergy, and Airway Symptoms, and a Systematic Review of Trends over Time for Childhood Asthma in Shanghai, China

**DOI:** 10.1371/journal.pone.0121577

**Published:** 2015-04-13

**Authors:** Chen Huang, Wei Liu, Yu Hu, Zhijun Zou, Zhuohui Zhao, Li Shen, Louise B. Weschler, Jan Sundell

**Affiliations:** 1 Department of Building Environment and Energy Engineering, School of Environment and Architecture, University of Shanghai for Science and Technology, Shanghai, PR China; 2 Tongji Architectural Design (Group) Co. Ltd. (TJAD), Shanghai, PR China; 3 Department of Environmental Health, School of Public Health, Key Laboratory of Public Health Safety, Ministry of Education, Fudan University, Shanghai, PR China; 4 161 Richdale Road, Colts Neck, New Jersey, 07722, United States of America; 5 Department of Building Science, School of Architecture, Tsinghua University, Beijing, PR China; University of Southern California, UNITED STATES

## Abstract

**Background:**

The prevalence of asthma among Shanghai children has increased over time. This increase might be associated with changes in environmental exposures. Investigation of the time-trend of asthma and current prevalences is essential to understanding the causes.

**Objective:**

To estimate the current prevalences of asthma, allergies and other respiratory symptoms among Shanghai preschool children, and to investigate the time-trend of childhood asthma prevalence of from 1990 to 2011.

**Methods:**

From April 2011 to April 2012, the CCHH (China, Children, Homes, Health) cross-sectional study was conducted in Shanghai. Questionnaires were distributed to 17,898 parents or guardians of preschool children from 72 kindergartens in 5 districts. Previous similar studies were also summarized by a systematic literature review.

**Results:**

From a total of 14,884 questionnaires for 3–7 year old children, prevalences of the following diseases and symptoms were calculated: asthma 10.2%, wheeze (ever) 28.1%, pneumonia (ever) 33.5%, otitis media 11.0%, rhinitis (ever) 54.1%, hay fever 12.2%, eczema (ever) 22.7%, and food allergy 15.7%. Urban children had higher prevalences of most symptoms than suburban children. The prevalence of asthma has increased significantly, almost five-fold, from 2.1% in 1990 to 10.2% in the present study. The prevalence of asthma in boys was higher than in girls in the present study and in all reviewed studies.

**Conclusions:**

Asthma, allergy and airway symptoms are common among preschool children in Shanghai. The prevalence of childhood asthma in Shanghai has increased rapidly from 1990 to 2011.

## Introduction

**Fig 1:**
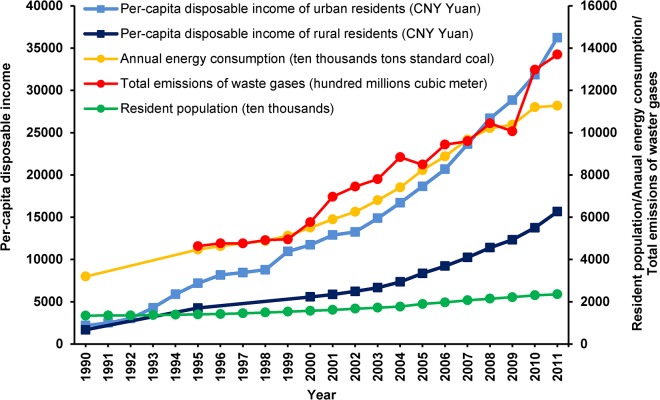
Indicators of rapid modernization in Shanghai from 1990 to 2011. Units for various indicators are showed in the brackets. All data obtained from Shanghai Statistical Yearbook 2012 [1]. Year-by-year data are displayed in the S1 Table.

During the period 1990–2011, Shanghai, one of the largest cities of China, rapidly modernized (Fig 1). The per-capita disposable incomes of urban residents and rural residents increased over sixteen-fold and nine-fold (calculated from data in S1 Table), respectively. More and more new-fashioned personal products, household appliances, building materials and furnishing materials have been used in residences [1, 2], changing indoor environmental exposures in Shanghai residences over the past 20 years [2, 3]. During the same time period in developed countries (Germany [4], Italy [5], Australia [6], and Switzerland [7]), where similar exposures to modern chemicals began many years ago and have plateaued, prevalences of asthma, allergies and airway symptoms among children seem to have reached a high plateau or even decreased. Meanwhile, in developing countries where home environments have recently and rapidly changed, prevalences of these diseases and symptoms among children appear to have been increasing [8, 9, 10, 11, 12]. Associations between specific environmental exposures and childhood respiratory health are still not clear and require further study. Since 1990, several studies have been conducted on childhood asthma, and related diseases or symptoms [13, 14, 15, 16, 17, 18, 19, 20] and their associations with ambient environmental exposure [21, 22, 23, 24, 25, 26] in Shanghai. But between 2000 and 2010, there has not been a large-scale study simultaneously conducted in urban and suburban districts of Shanghai city. Thus it is of interest to investigate to what extent childhood asthma prevalence has increased in this rapidly developing city.

The present paper is part of the epidemiological survey (CCHH: China, Children, Homes, Health) of 10 Chinese cities [27]. It aims to: 1) report the current prevalences of childhood asthma, allergies and other respiratory symptoms in Shanghai; 2) review and summarize all previous related studies to describe the time-trend of childhood asthma prevalence in Shanghai. S2 Table shows PRISMA (Preferred Reporting Items for Systematic Reviews and Meta-Analyses) checklist for the systematic review.

## Materials and Methods

### Ethics Statement

The questionnaire and detailed proposal for the CCHH study in Shanghai were approved by the ethical committee in the School of Public Health, Fudan University, Shanghai, China (International Registered Number: IRB00002408&FWA00002399). We thoroughly informed potential participants of the purpose, proposal details and potential concerns of the study by oral presentation or written explanation. All participants verbally consented for themselves and for the preschool children for whom they responded to questionnaires. All participants voluntarily responded to the survey. The ethical committee approved this procedure for obtaining consent.

### The CCHH study

The CCHH study consists of two phases, a cross-sectional study (phase one: questionnaire) and a nested case-control study (phase two: indoor-testing, home-inspections and sample collecting and analyzing). Its focus is associations between household environmental exposures and asthma, allergy and other respiratory symptoms among preschool children. Since 2010, the cross-sectional study has been conducted in ten large cities in mainland China [25]. Shanghai is one of these cities. Phase one of CCHH (questionnaire survey) in Shanghai was performed from April 2011 to April 2012.

### Study population and Questionnaire

**Fig 2:**
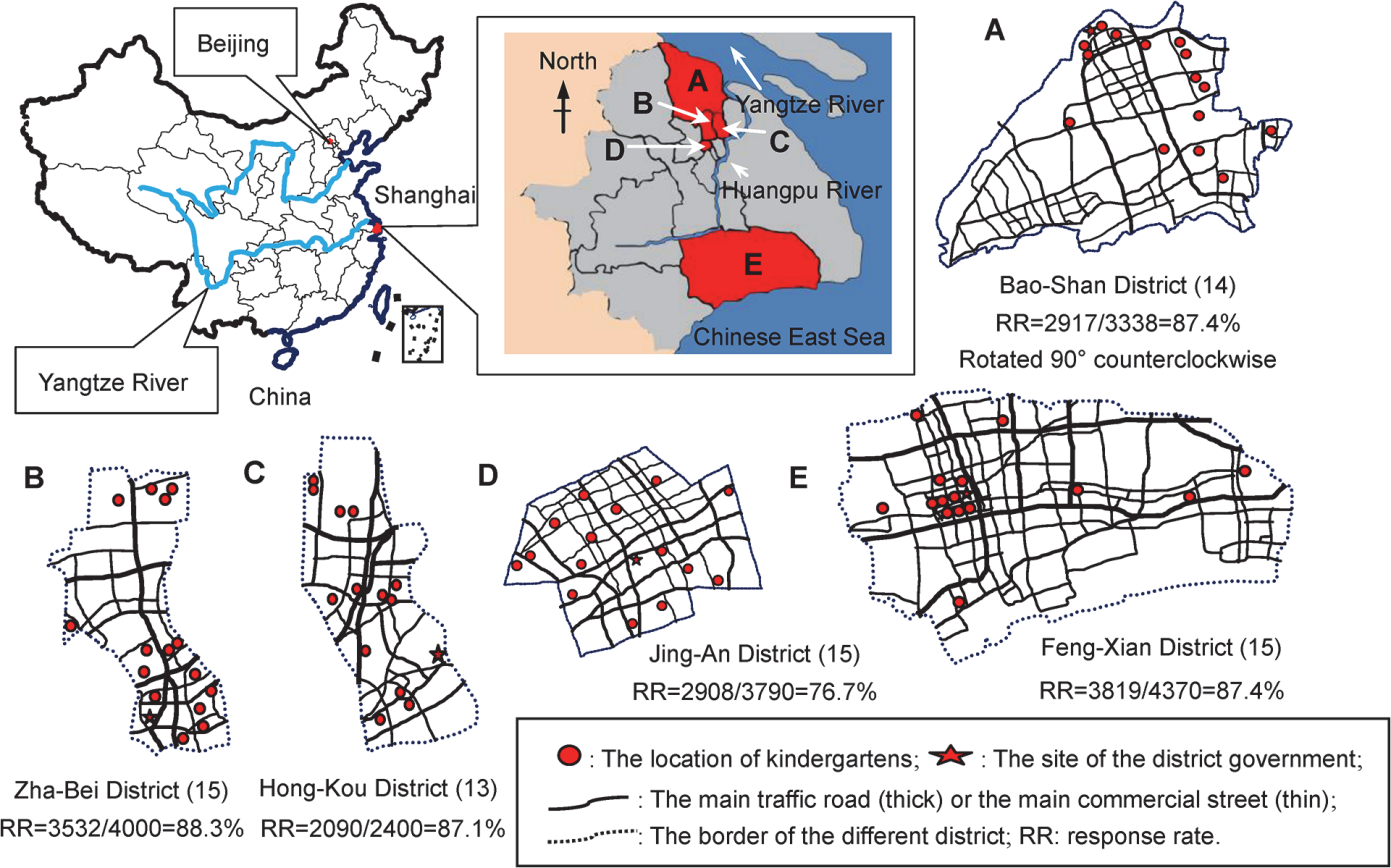
Distribution of kindergartens in this survey. The number in the bracket is the number of kindergartens we have surveyed in the district. Because most of the kindergartens in Feng-Xian District (E) are located in the center of district, the kindergartens we surveyed in this district are relatively centralized.

Shanghai, located in the Yangtze River estuary, had a population of more than 23 million in 2011 [1] including 1.07 million preschool children (< 6 years old) [28]. Our cross-sectional study surveyed preschool children in kindergartens. To obtain a representative sample, a multistage hierarchical sampling method was used. Firstly, in 2010 three urban districts (Jing-An, Zha-Bei, and Hong-Kou) and two suburban districts (Feng-Xian and Ban-Shan) from 18 districts of Shanghai (8 urban districts and 10 suburban districts) were selected. Then, about 15 kindergartens in each district were randomly chosen (Fig 2). A total of 17898 parents of preschool children from 72 kindergartens in these districts were surveyed.

The core questions about diseases were essentially the same as in the ISAAC (International Study of Asthma and Allergies in Childhood) study [29]. The questions about family background, infant exposure, building characteristics, surrounding environment, and lifestyle are similar to those used for the DBH (Dampness in Buildings and health) study in Sweden [30], the ALLHOME study in Bulgaria [31] and the CCHH studies in other Chinese cities [27]. All questions were translated into Mandarin. Some questions have been adapted to fit better with Shanghai aspects of culture and lifestyle. Questions for diseases and symptoms are presented in S3 Table of the present study and in the supplemental information of reference [27]. To conduct the survey, we preliminarily contacted the administrations of the kindergartens in the Shanghai Municipal and District Bureau of Education and presented the study and questionnaires to the kindergarten leaders. We included all kindergarten children in any school or class we visited and recommended that the child’s mother fill out the questionnaire. Questionnaires were distributed in two ways: 1) In urban districts, our members firstly described the questionnaires at parent-teacher meetings, then gave them to parents and recovered them on-site or later from the kindergarten children; 2) In suburban districts, our members distributed the questionnaires to teachers, who distributed them via children to parents and collected the completed questionnaires from the children.

### Literature search strategy

The literature search was conducted in CKNI (Chinese Knowledge National Infrastructure) and VIP (Database for Chinese Technical Periodicals) in Chinese, and PubMed and Web of Science in English. In CKNI and VIP, we used “asthma” (in the title or keywords), “child” or “infant” (in the title or keywords), “Shanghai” or “China” (in the abstract) as search terms. In PubMed and Web of Science, we used “asthma”, “children” or “infant”, “Shanghai” or “China”, and “prevalence” or “incidence” (in title or abstract) as search terms. We identified 275 records identified after removing duplicates from January 1990 to October 2012.

### Inclusion criteria

**Fig 3:**
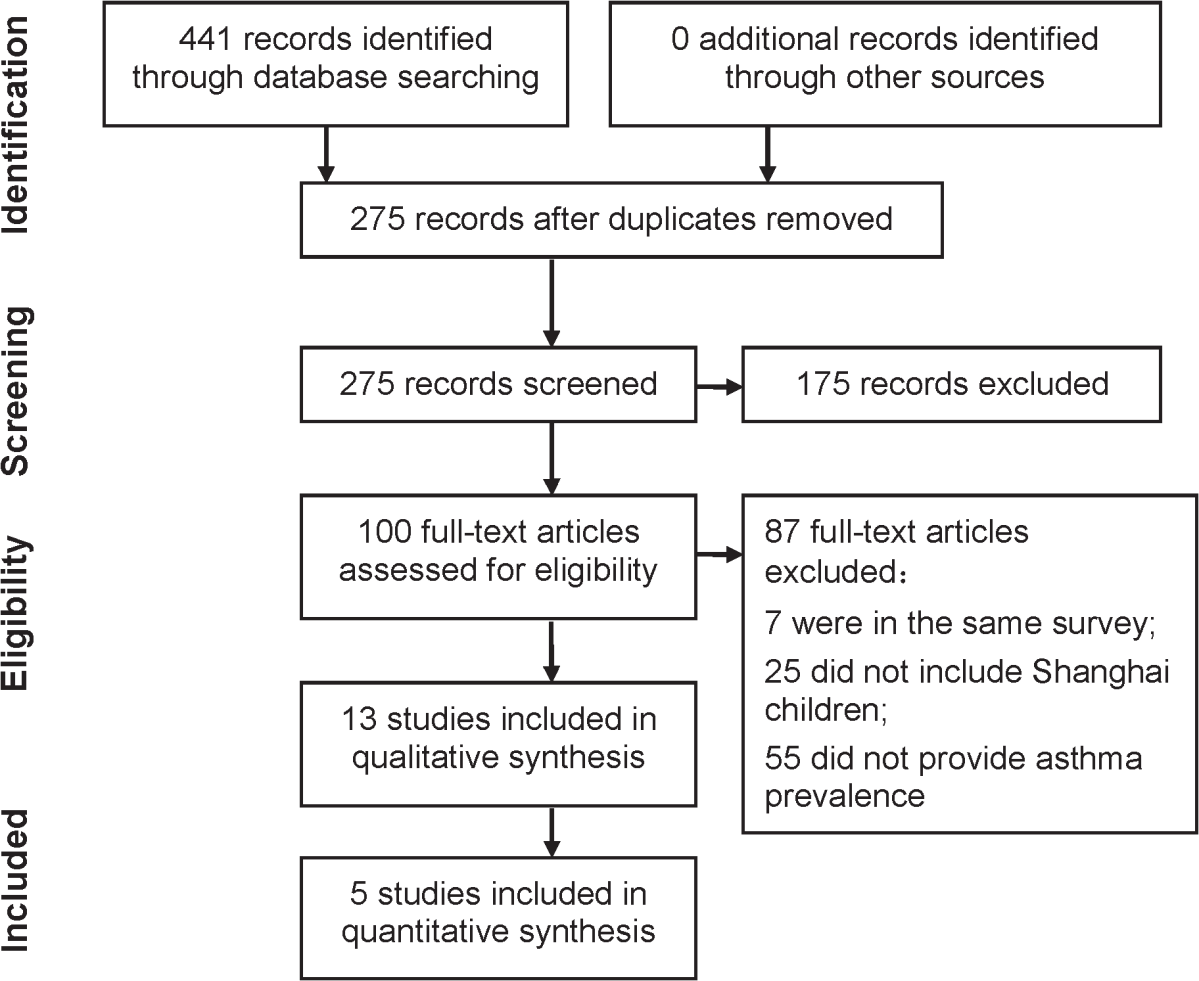
Flow chart of information of literature search through different phases (PRISMA flowchart).

Those cross-sectional or cohort studies which reported prevalence of childhood asthma in Shanghai were included in the qualitative synthesis. Thirteen studies were finally selected according to the inclusion criteria. Herein 12 studies were in Chinese, and one in English. All of these studies were cross-sectional studies and based on cluster sampling and questionnaires. Then, those studies that met the following criteria were included in the quantitative synthesis: 1) the study was conducted all over Shanghai city (included urban and suburban districts); 2) the study provided sample numbers with sufficient detail for us to calculate the prevalence of asthma among children in different age groups; 3) the study used the same ISAAC questionnaire [29] or a similar questionnaire, as our CCHH study [27]. Finally, five studies [13, 16, 18, 19, 23] were selected to analyze the time trend of childhood asthma prevalence in different districts and within different age groups, including infancy (0~2 years old), preschool (3~7 years old), school (8~12 years old) and puberty (13~14 years old). Herein, two studies that included 3–7 years old preschool children and were conducted in 1990 [13] and in 2000 [16] were selected to analyze the time trend for the specific ages 3, 4, 5, 6, and 7 years old. Details of data in these studies are shown in the S4 and S5 Tables. Fig 3 shows the flow of information through the selected phases (PRISMA flowchart).

### Data-selection and Statistical Analyses

After the questionnaires were recovered, they were carefully checked and coded before data-inputting. Questionnaires were excluded if more than thirty percent of the items had not been filled out. Then data in valid questionnaires were inputted by trained master students. Each database was built by one student and checked twice by two group members using random sampling before it was added to the Shanghai overall databases. The data regarding childhood diseases and symptoms, but not the data about home environmental factors and family lifestyles, were analyzed in the present study. Due to missing data for various items, the sums of children with different diseases in subgroups are not necessarily equal to the total numbers.

SPSS version 17.0 (SPSS Ltd., Chicago, Illinois, USA) was primarily used for analyses. Pearson’s chi-squared (χ^2^) test was used to test for significance of difference in prevalences between different children groups. A *p*-value < 0.05 indicated statistical significance. Data extracted from selected publications were used to calculate the prevalence of asthma in different age groups. Microsoft Excel 2010 (Microsoft Ltd. Seattle, Washington, USA) was used for these calculations, as well as to make scatter plots, trend lines and to calculate equations for asthma prevalences versus time in an exponential regression model.

## Results

### Basic data and current prevalence

A total of 15,266 questionnaires from children aged 1–8 years were valid. The response rate was 85.3%. Very few children aged 1, 2 and 8 year old attend kindergarten in Shanghai; accordingly, very only 23, 10, and 49 questionnaires respectively were obtained for these age groups. Questionnaires for these age groups as well as 300 additional questionnaires that did not report the child’s age were excluded. A total of 14,884 questionnaires for children aged 3–7 years were selected for further analyses. Herein 70.1% of the questionnaires were answered by the children’s mothers. Boys’ and girls’ questionnaires account for 50.8% and 49.2% respectively.

Table 1 shows the current prevalences of different symptoms and diseases among 3–7 year old children in Shanghai. Prevalences of asthma, wheeze (ever), pneumonia, rhinitis (ever), hay fever, eczema (ever), and otitis media were 10.2%, 28.1%, 33.5%, 54.1%, 12.2%, 22.7%, and 11.0%, respectively. Boys’ prevalences were significantly higher than girls’ except for dry cough, eczema (in the last 12 months), and otitis media. Children in urban kindergartens had higher prevalences than those in suburban kindergartens except for wheeze, dry cough, and common cold (≥ 3 times) in the last 12 months (Table 1 and S1 Fig). Three year old children had the highest prevalence of eczema (in the last 12 months); four year old children had the highest prevalence of multi-episodes (≥ 3 times, in the last 12 months) of common cold; five year old children had the highest prevalences of wheeze, dry cough, pneumonia, and hay fever; and six year old children had the highest prevalence of asthma (11.4%). Prevalences of dry cough and rhinitis (in the last 12 months), significantly decreased in children older than five years old, while prevalences of eczema (in the last 12 months) decreased with increasing age.

**Table 1:** Prevalences of asthma, allergy and airway symptoms tabulated by gender, residential district and age (3~7 years old), *n* (%).^a^

	Total	Sex		Urban or Suburban area		Age (years)				
Diseases or symptoms	(*N* = 14884)	M(*N* = 7536)	F (*N* = 7296)	U(*N* = 8353)	S(*N* = 6531)	3(*N* = 749)	4(*N* = 5561)	5(*N* = 4399)	6(*N* = 3375)	7(*N* = 800)
Wheeze, ever	4110 (28.1)	2298 (31.0)	1820 (25.1)***	2310 (28.1)	1800 (28.0)	190 (26.0)	1495 (27.3)	1271 (29.3)	956 (28.8)	198 (25.3) *
Wheeze in last 12 months	3163 (21.6)	1747 (23.5)	1410 (19.6)***	1736 (21.1)	1427 (22.1)	155 (21.2)	1197 (21.8)	951 (21.9)	710 (21.3)	150 (19.1)
Dry cough in last 12 months	2855 (19.4)	1419 (19.0)	1426 (19.8)	1647 (19.9)	1208 (18.7)	162 (22.0)	1175 (21.3)	853 (19.6)	541 (16.2)	124 (15.7)***
Doctor diagnosed asthma ^b^	1471 (10.2)	871 (11.9)	597 (8.4)***	952 (11.7)	519 (8.2)***	54 (7.4)	500 (9.2)	468 (11.0)	374 (11.4)	75 (9.7)**
Croup, ever	1069 (7.5)	608 (8.5)	457 (6.6)***	648 (8.1)	421 (6.8)**	54 (7.6)	378 (7.1)	330 (7.9)	255 (7.9)	52 (6.8)
Pneumonia, ever	4815 (33.5)	2503 (34.4)	2297 (32.5)*	2846 (35.1)	1969 (31.4)***	226 (31.2)	1720 (31.9)	1480 (34.9)	1135 (34.8)	254 (33.1) **
Rhinitis, ever	7893 (54.1)	4130 (55.9)	3735 (52.2)***	4822 (58.9)	3071 (48.0)***	424 (58.3)	2912 (53.3)	2345 (54.6)	1819 (54.9)	393 (50.3) *
Rhinitis in last 12 months	6142 (42.5)	3236 (44.1)	2889 (40.7)***	3854 (47.2)	2288 (36.3)***	345 (47.3)	2329 (42.9)	1834 (42.9)	1369 (41.9)	265 (34.4)***
Rhinitis on pet exposure ^c^	592 (4.6)	336 (5.1)	253 (4.0)**	397 (5.2)	195 (3.6)***	28 (4.2)	210 (4.3)	172 (4.5)	150 (5.2)	32 (4.6)
Rhinitis on pollen exposure ^c^	1015 (7.7)	567 (8.6)	447 (6.9) ***	762 (9.9)	253 (4.6)***	42 (6.3)	362 (7.3)	325 (8.4)	245 (8.4)	41 (5.9) *
Doctor diagnosed hay fever ^b^	1765 (12.2)	1032 (14.1)	728 (10.3)***	1192 (14.7)	573 (9.0)***	59 (8.0)	624 (11.6)	582 (13.7)	418 (12.8)	82 (10.6)***
Eczema, ever	3160 (22.7)	1609 (22.9)	1535 (22.5)	2095 (26.7)	1065 (17.6)***	176 (25.6)	1256 (24.0)	924 (22.5)	676 (21.6)	128 (17.2)***
Eczema in last 12 months	1882 (13.1)	927 (12.8)	945 (13.4)	1250 (15.5)	632 (10.1)***	121 (16.9)	770 (14.3)	540 (12.8)	385 (11.9)	66 (8.5) ***
Food allergy, ever	2242 (15.7)	1168 (16.1)	1068 (15.3)	1403 (17.5)	839 (13.3) ***	120 (16.7)	853 (15.9)	671 (15.8)	490 (15.2)	108 (14.2)
Otitis media, ever	1592 (11.0)	789 (10.7)	794 (11.2)	962 (11.8)	630 (9.9)***	56 (7.7)	510 (9.3)	531 (12.4)	402 (12.3)	93 (12.0) ***
Common cold (≥ 3 times) ^c^	6103 (43.1)	3117 (43.5)	2974 (42.8)	3217 (40.4)	2886 (46.5)***	311 (43.5)	2579 (48.1)	1782 (42.5)	1188 (37.4)	243 (33.5) ***

^a^ “*n*” is the number of children who had asthma; “*N*” is the total number of children in different groups; M: Male; F: Female; U: Urban area; S: Suburban area.

^b^ during lifetime since birth (ever);

^c^ in last 12 months (before questionnaire);

* 0.01≤*p*<0.05

**0.001≤*p*<0.01

***p<0.001 in Pearson’s chi-squared test.

**Fig 4:**
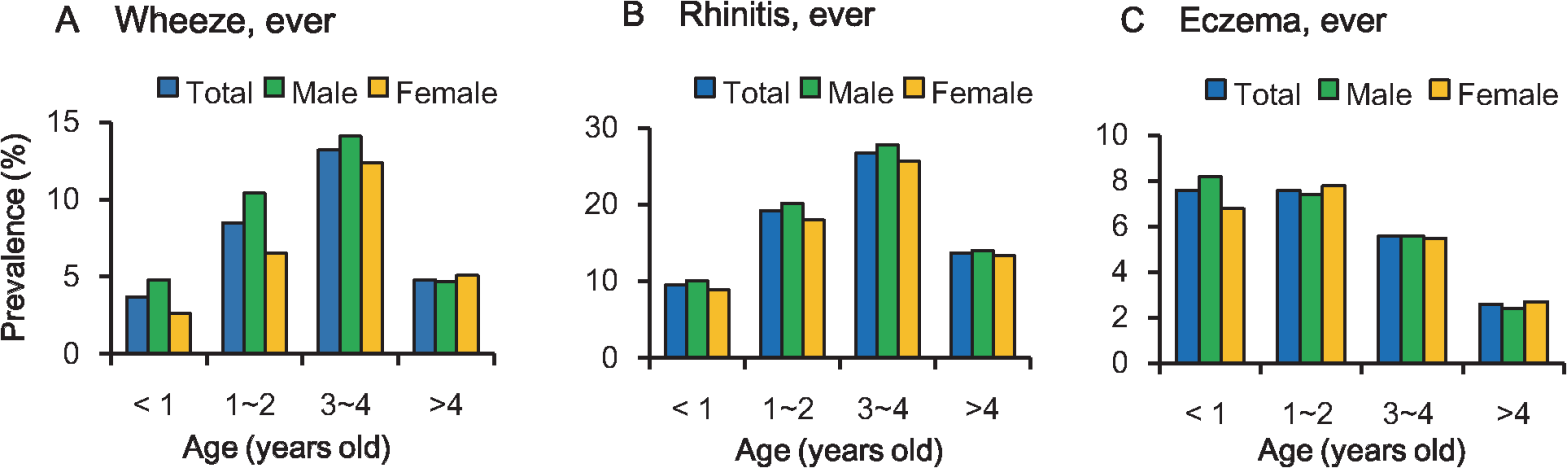
Prevalences of wheeze, rhinitis, and eczema (ever) at different ages during lifetime since birth. Detailed data for prevalences in the S6 Table.

Fig 4 shows the prevalences of wheeze, rhinitis, and eczema for each age group. The highest prevalences for wheeze and rhinitis symptoms were for 3–4 year olds, whereas the highest prevalence of eczema symptoms was for < 1 and 1–2 year olds. More girls than boys at 1–2 and > 4 years old reported eczema. More boys than girls < 4 years old reported wheeze, but more girls than boys > 4 years old reported wheeze. More boys than girls at all ages reported rhinitis symptoms.

Table 2 shows the prevalences of food allergies. The four most common allergenic foods for preschool children in Shanghai were sea food, eggs, milk or dairy products, and fruit. There were no significant differences between boys’ and the girls’ prevalences of food allergies. However, urban children had significantly higher prevalences of food allergies for the four most common allergenic foods and nuts than suburban children.

**Table 2:** Prevalences of food allergies.

		Sex		Districts	
Category of food	Total	Male	Female	Urban	Suburban
Sea food	1198 (10.1)	628 (10.5)	567 (9.8)	767 (11.8)	431 (8.1) ***
Eggs	362 (3.1)	190 (3.2)	171 (3.0)	246 (3.8)	116 (2.2) ***
Milk or dairy Products	295 (2.5)	152 (2.5)	143 (2.5)	209 (3.2)	86 (1.6) ***
Fruit	245 (2.1)	120 (2.0)	123 (2.1)	155 (2.4)	90 (1.7) **
Nuts	85 (0.7)	47 (0.8)	37 (0.6)	64 (1.0)	21 (0.4) ***
Meat	60 (0.5)	33 (0.6)	27 (0.5)	36 (0.6)	24 (0.5)
Bean	50 (0.4)	26 (0.4)	23 (0.4)	34 (0.5)	16 (0.3)
Vegetables	37 (0.3)	20 (0.3)	17 (0.3)	14 (0.3)	23 (0.4)
Flour	12 (0.1)	5 (0.1)	7 (0.1)	6 (0.1)	6 (0.1)

**0.001≤*p*<0.01

***p<0.001 in Pearson’s chi-squared test.

### Time-trends in the prevalence of asthma

Table 3 summarizes basic information from those previous studies that were included in the qualitative analysis. Most of these studies included preschool children (< 8 years old). The most frequently used method to obtain information was questionnaires responded to by parents at home or at school. Definitions of the prevalence of asthma and sample size varied among studies. The 1990 study did not provide the response rate, but all other studies reported very high response rates. The overall prevalence of asthma among 0–14 year old children in 1990 was 1.79% [13], but was 10.2% among 3–7 year old children in the present study.

**Table 3:** Basic information for studies which were included in the qualitative analysis of time-trend prevalence of asthma.

Year [Ref.]^a^	Age (years)	Location (District)^b^	Method for questionnaire	Reference questionnaire^c^	Definition of asthma prevalence	Valid sample (*N*)	Response rate (%)	P(%)^d^
1990 [13]	0~14	BS, CN, HK, HP, JA, LW, XH, YP, ZB	Home, parents-reported	NP	Diagnosed asthma, ever (Cumulative incidence)	38288	NP	1.79
1994 [14]	13~14	Shanghai^e^	School, students-reported	ISAAC [29]	History of diagnosed asthma (Cumulative incidence)	3483	99.00	7.10
1997 [15]	0~10	Jin-Qiao area in Pu-Dong District	Home, parents-reported	NP	Diagnosed asthma, ever; rechecked by three doctors in three different hospitals	5920	100.00	0.51
2000 [16]	0~14	BS, HK, HP, LW, PT, XH	Home, parents-reported	ISAAC [29]	Diagnosed asthma, ever (Cumulative incidence)	14462	98.74	4.52
2000 [21]	13~14	Shanghai^e^	School, students-reported	ECRHS [32]	Diagnosed asthma, ever (Cumulative incidence)	1414	99.00	8.90
2005 [22]	6~13	Shanghai^e^	School, parents-reported	ISAAC [29]	The child has had asthma in the last year (Current)	4395	92.50	7.20
2006 [17]	6~9	Jin-Yang Community in Pu-Dong District	School, parents-reported	NP	Diagnosed asthma, ever (Cumulative incidence); The case history was rechecked	4895	95.14	2.25
2006 [23]	6~14	LW, QP, YP, ZB	School, parents-reported	NP	Diagnosed asthma, ever (Cumulative incidence)	7126	100.00	5.92
2007 [18]	4~17	Urban area^e^	School, parents or students reported	AST [33]	1) Wheezing with more than two episodes acute dyspnea; or 2) diagnosed asthmatic bronchitis or infant asthma; or 3) wheezing with dyspnea	6551	90.60	4.50
2008 [19]	0~19	Pu-Tuo District	School or medical center, parents or children-reported	NP	History of wheezing or diagnosed asthma (Cumulative incidence)	11771	99.46	7.79
2008 [20]	3~7	Pu-Tuo District	School, parents-reported	NP	Diagnosed asthma, ever; rechecked by a specialist	1554	100.00	6.63
2009 [24]	7~8	JD, LW	Home, parents-reported	NP	Diagnosed asthma, current; rechecked by 4 specialists	1511	100.00	7.68
2010? [25]	4~14	BS	Home, parents-reported	NP	Diagnosed asthma, ever; rechecked by a specialist	257	99.12	3.89
2011	3~7	BS, FX, HK, JA, ZB	School, parents-reported	ISAAC [29]	Diagnosed asthma, ever (Cumulative incidence)	14884	85.30	10.20

^a^ Year when the survey was done, not when the article was published; Ref.: Reference; “?”: year was inferred, not provided in the references.

^b^ BS: Bao-Shan, CN: Chang-Ning, HK: Hong-Kou, HP: Huang-Pu, JA: Jing-An, JD: Jia-Ding, LW: Lu-Wan, XH: Xu-Hui, QP: Qing-Pu, YP: Yang-Pu, ZB: Zha-Bei.

^c^ AST: American Thoracic Society; ECRHS: European Community Respiratory Health Survey; “NP” stands for that the information is not provided in the literature.

^d^ Prevalence;

^e^ The specific district was not provided in the reference.

**Fig 5:**
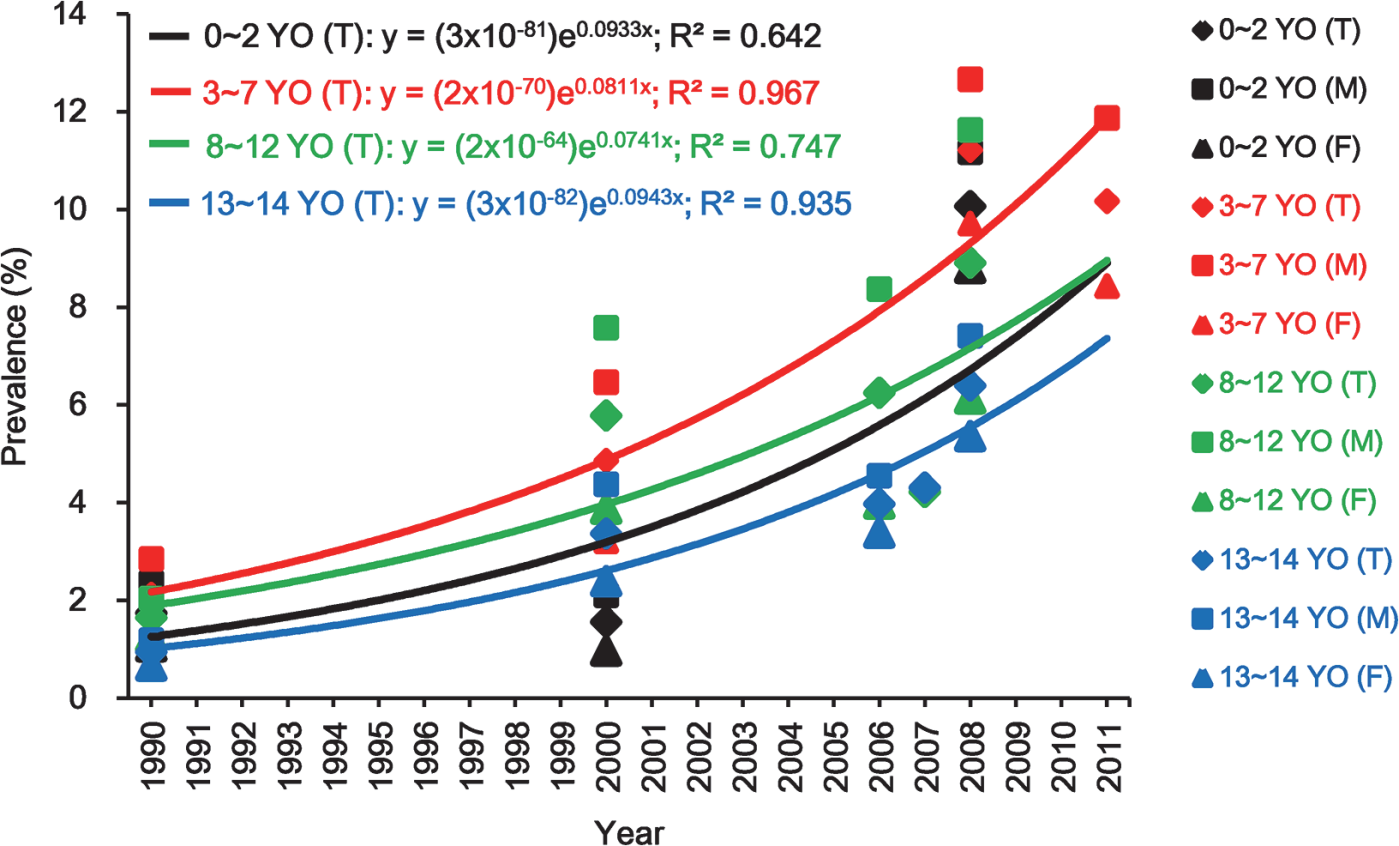
Time-trend prevalences of asthma among different age children from 1990 to 2011. Herein, “YO” stands for “years old”. T: Total; M: Male; F: Female. The solid lines are exponential trend lines. Prevalences for 2008 include wheezing as well as asthma ever. Detailed data for prevalences are in the S7 Table. Reference: 1990 [13]; 2000 [16]; 2006 [23]; 2007 [18]; 2008 [19].

**Fig 6:**
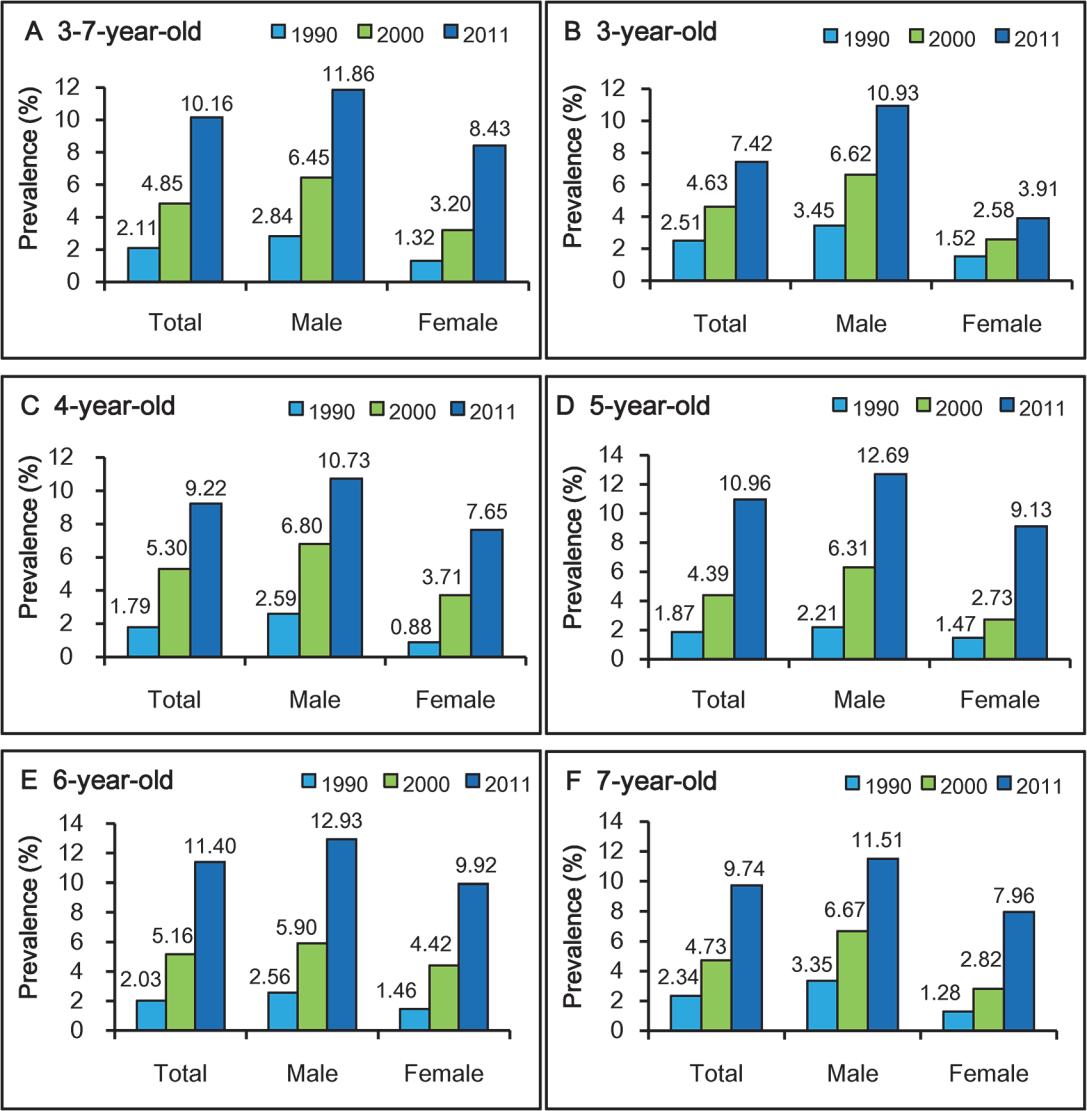
Time-trend prevalences of asthma among 3–7 year old children from 1990 to 2011. References: 1990 [13]; 2000 [16].

Calculated prevalences of asthma in different age groups (infancy, preschool age, school age, and puberty) for the past 22 years are shown in Fig 5 and S7 Table. Prevalences have clearly increased from the 1990s, although the prevalences among 8–12 year old children have fluctuated. Prevalences in different age and sex groups showed significant differences. Relative to 0–2 year old and 13–14 year old children, 3–7 year old children had significantly higher prevalence. In 1990, the prevalence among 3–7 year old children was 2.11%, while in our survey it had grown almost five-fold to 10.2%. For 3–7 year old children, boys’ prevalence has increased more than four-fold from 2.84% in 1990 to 11.86% in 2011, while girls’ prevalence has increased more than six-fold from 1.32% in 1990 to 8.43% in 2011. The solid line in the Fig 5 shows an exponential uptrend in prevalence. The 3–7 year old age groups had the highest, and the 13–14 year old age groups had the lowest inferred prevalences respectively. Furthermore, Fig 6 shows the time-trend of prevalences among 3, 4, 5, 6 and 7 year old children. Prevalences in different age and sex groups show significant differences. Prevalences for both boys and girls consistently had a notable uptrend in each age group, and all boys’ prevalences are higher than girls’.

The time-trend for prevalences of asthma in different districts of Shanghai is also shown in S2 Fig. Similar surveys have been conducted in most districts of Shanghai; only Chong-Ming Country, Ming-Hang District and Jing-Shan District have not been surveyed. In districts that have had more than two surveys (nine districts of fifteen), all prevalences have significantly increased. The urban districts (A-I in the S2 Fig) have always had higher prevalences than suburban districts. In 1990, Huang-Pu District had the highest prevalence (2.86%). The highest prevalence (14.5%) was in Hong-Kou District in 2011.

## Discussion

In this study, current prevalences of asthma and other symptoms or diseases among preschool children in Shanghai are reported and analyzed. Compared to prevalences in other Chinese cities, where similar surveys have been conducted in recent years, 3–7 year old children in Shanghai had the highest prevalence of asthma (10.2%) [10, 12, 27, 34]. A cross-sectional survey among 0–14 year old children conducted in Beijing, Chongqing and Guangzhou from October 2008 to May 2009, found that the highest prevalence of asthma in these three cities was 7.45% in Chongqing children [10]. The prevalences among 3–6 year old children in the parallel CCHH studies in Beijing and Chongqing were 6.1% and 8.2%, respectively, in 2011 [12, 27]. The prevalence of asthma among 6–13 year old children in Liaoning Province, northeast China, was estimated at 5.8% in 2007 [34].

The Shanghai prevalence of asthma among preschool children also is comparable with those reported for several other countries, for example, Italy [5], Australia [6], Korea [35, 36], and Sweden [37]. ISAAC studies have found that the prevalence of asthma among 6–7 and 13–14 year old children ranged from 2%-4% (the lowest) in Albania, Indonesia, and Romania to 30%-32% (the highest) in Costa Rica, New Zealand, and the United Kingdom in 2002 [9, 38]. The global prevalences of asthma among 6–7 and 13–14 year old children were estimated at 14.1% and 11.7%, respectively in 2002 [39].

The present study also reports previous and current prevalences of asthmatic symptoms, allergies, and airway symptoms. Thus, in Shanghai, the prevalence of wheeze symptoms (ever) was 6.7% among 13–14 year old children in 1994 [14], 7.1% among 4–17 year old children in 2007 [18], but 28.2% among 3–7 year old children in the present study. The prevalence of wheeze (in the last 12 months) was 3.3% among 13–14 year old children in 1994 [14, 40] but 21.6% in 3–7 year old children in 2011 as reported in the present CCHH study.

The prevalence of rhinitis symptoms (ever) is twice as large, 54.2% compared to 26.8%, for preschool children in the present study as for the 13–14 year old Shanghai children in 1994 [14]. The prevalence of rhinitis symptoms (in the last 12 months) is also twice as large, 42.5% compared to 21.6%, in the present 2011 Shanghai children as in the 1994 children [14]. These current prevalences of rhinitis (ever and in the last 12 months) are higher than those reported in a 2002 ISAAC phase three study of Beijing and Hong Kong 13–14 year old children, and for Hong Kong and Taipei 6–7 year old children [41].

The current Shanghai prevalence of doctor-diagnosed hay fever (allergic rhinitis) (12.3%) is higher than the 9.3% reported for 13–14 year old children in 1994 [14] and the 7.44% reported for 3–17 year old children in 2007 [42]. It is also higher than those reported for other Chinese cities, such as Beijing (7.6%), Guangzhou (4.1%), and Hong Kong (1.5%), but lower than for 13–14 year old children in Taipei (33.5%) in 2002 [43]. It is higher than for Hong Kong 6–7 year old children (1.5%) but lower than for Taipei 6–7 year old children (39.2%) as reported in the ISAAC phase III study in 2002 [43].

The prevalence of eczema (ever, 22.7%) in the present study was higher than for 0–14 year old children in Guangzhou (7.22%), Chongqing (10.0%), and Beijing (20.6%) in 2008 [18]. It was comparable with eczema rates reported for many other counties or regions in a 1999 ISAAC study [44], but slightly lower than the 24.6% reported for 1–8 year old children in Seoul, Korea in 2009–2010 [36]. The 33.5 percent of children for whom lifetime-ever pneumonia was reported in Shanghai was higher than for all CCHH cities except for the 38.1% reported for Changsha [27].

In the CCHH cities summary, Zhang et al [27] published current prevalences of asthma, allergies, and airway symptoms among preschool children in ten Chinese cities. More comparisons of current prevalences for these cities have also been presented in this article [27]. From the Zhang et al. summary combined with the present analysis, we note that current Shanghai prevalences of asthma, allergy and related symptoms are the highest or among the highest for the CCHH cities. This parallels economic development level and status, which for Shanghai is greater than the other cities in China [1, 45]. Therefore, it could possibly be inferred that the increase in childhood asthma and related diseases and/or symptoms in Shanghai is associated with the rapid economic growth and/or modernization [2, 3].

We compared the prevalence of asthma in Shanghai 3–7 year old children, the largest age group in our study, with prevalences reported for Shanghai from 1990 to 2010. Using data published in two of these studies [13, 16], we have calculated asthma prevalences for comparison among 3, 4, 5, 6, and 7 year old children integrally and separately (Fig 6). In particular, we note that the prevalence among 6 year old children, in whom asthma is more reliably diagnosed than in younger children [46, 47], has increased from 2.03% in 1990 [13] to 11.4% in 2011 (Fig 5). Based on these analyses, we infer that the prevalence of childhood asthma in Shanghai has greatly increased from 1990 to 2011. The parallel CCHH study in Beijing [12] also has shown a similar uptrend curve for asthma prevalence among preschool children. It is unlikely that asthma prevalence can increase to where all of the population has asthma, because it is likely that a genetic predisposition is necessary for the development of asthma [48]. We can speculate that the asthma prevalence among different age groups that are presently growing linearly will level at a plateau value as has happened in other countries [4, 5, 6, 7]. The upper limit for prevalence can be estimated at somewhat >30% from those countries whose prevalence have decreased or plateaued [4, 5, 6, 7, 9]. Presently, asthma prevalence among children in Shanghai appears to be in the region of steep increase in a sigmoid curve as in Beijing [12]. However, at its present 10.2%, it is far short of the potential maximum of 30%. Thus, if environmental risk factors could be identified and changed, prevalence at a lower plateau could possibly be attained.

In the present study, prevalences did not have perfectly consistent trends (Table 3). For example, prevalence among 8–12 year old children in 2007 [18] was lower than in 2006 [23] and in 2008 [19]. There are several possible explanations for these inconsistencies. Firstly, most of the cited surveys were conducted in relatively few districts with small sample sizes. Only the surveys conducted by The Cooperation Group on Childhood Asthma of Shanghai Medical Association (CGCASMA) in 1990 [13] and 2000 [16], and our survey in 2011–2012 were conducted in more than five districts, included both urban and suburban districts, and had large sample sizes (Table 3). We note that the present study, with a relatively large sample size, shows significantly higher prevalences of asthma and asthmatic symptoms as well as lifetime-ever incidence of pneumonia in urban than suburban areas. Secondly, methods used to diagnose asthma in these surveys may have differed, and studies for comparison may have used different methods or questions in the questionnaires. For example, asthma diagnoses in Hang et al.’s study (1997) [15] were rechecked by three doctors in three different hospitals simultaneously, while in Zhang et al.’s study (2008) [19], asthma was defined as a history of either wheezing symptoms or doctor-diagnosed asthma ever. It is likely that the prevalences reported in the 1997 study [15] are more reliable than those of the 2008 study [19] which may have overestimated asthma prevalence. Thus, inconsistencies in asthma prevalences between 1994, 2008 and 2011 are likely due in part to methodological differences in the studies. By necessity, systematic reviews of asthma time-trends [11, 49, 50, 51] include studies with distinctly different study populations, questionnaires, and aims, and combined them to create an approximate time series of prevalence. We think it likely that the strength of the upward asthma prevalence trend over the past 22 years is real in spite of the methodological differences among the studies we used. Thirdly, a strengthening economy and increased wealth in Shanghai over the past 20 years, makes it likely that both the technical ability to diagnose asthma and parents’ desire to bring sick children to the doctor has increased. These changes suggest that the present study reports more actual prevalences whereas earlier reports may have underestimated prevalences. However, among the CCHH cities whose economic development in the same time period has been less than that of Shanghai [45], asthma prevalences have also increased [27]. For Shanghai, both previous studies and the present study have found significantly higher asthma in urban areas than in suburban areas, consistent with other studies [11, 24, 35, 38]. Also, both previous studies and the present study have found significantly higher asthma prevalences in boys than girls, consistent with many Chinese [10, 11, 37, 38] and international [4, 5, 6, 7, 9, 35, 36] studies. Young boys have a higher asthma prevalence than girls at least until age 13–14 [52] or adolescence [53, 54].

We acknowledge that there may be bias because of self-reported symptoms and exposures by the children’s parents or guardians. This bias is mitigated in the present study by a large sample size with a high response rate. The time-trend of childhood asthma among 3–7 year old children is most likely well characterized. The present comprehensive description of asthma, allergy, and airway symptoms or diseases in preschool Shanghai children with similar demographic factors, and the summary of childhood asthma epidemiological studies conducted in Shanghai in the past 22 years (Table 3). It also provides a good reference for national or international studies and comparisons of childhood health issues which will be useful for future similar studies in rapidly developing cities, countries, or regions.

**Fig 7:**
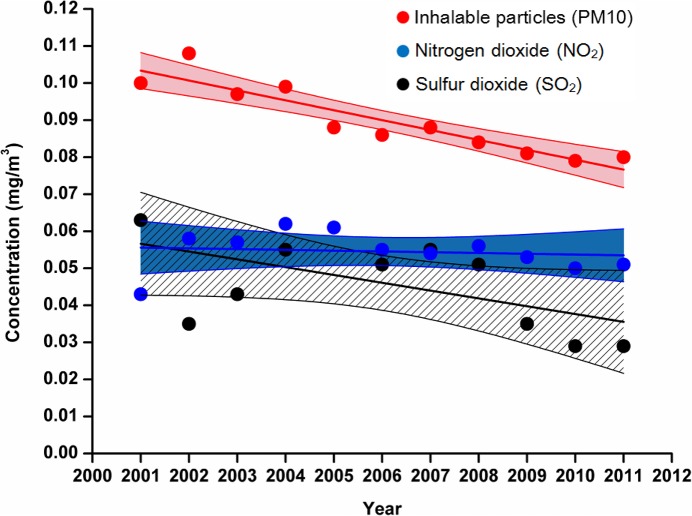
Time trend of outdoor air pollutant concentrations in Shanghai from 2001 to 2011. Linear Trend Lines and their 95% confidence regions are also displayed. Data obtained from Shanghai Environment Yearbooks 2002–2012. Detailed data and references are displayed in the S8 Table.

Furthermore, given the parallel exponential uptrends of prevalences of childhood asthma in different age groups (Fig 5) and of various indicators of rapid modernization in Shanghai (Fig 1), we can ask whether the disease increase is associated changes in environmental exposures (indoor and outdoor) and family lifestyles that have occurred with rapid modernization [2, 3]. However, with respect to air pollution, while many studies have found significant associations between air pollution and childhood asthma and other respiratory diseases and/or symptoms [55], the annually averaged concentrations of typical outdoor air pollutants in Shanghai (Fig 7) have trended down from 2001 to 2011. Thus, it is possible that environmental exposures whose sources are indoors rather than outdoors may have stronger associations with childhood asthma than outdoor air pollution [2, 3, 21, 22, 23, 24, 25, 26, 30, 34]. We have previously reported that some environmental factors, including pet-keeping [56], indoor tobacco smoking [57], using wood as cooking fuel, living within 200m of a highway or busy road [58], and home dampness-related indicators [59], have positive and significant associations with childhood asthma and other diseases or symptoms. We have been conducting the case-control study (Phase II of the CCHH study) since March 2013 in Shanghai, and anticipate finding more associated environmental factors.

## Conclusions

The prevalence of asthma among Shanghai children at different ages and for both genders has increased considerably in the past 22 years. More attention should be given to the health effects of changing environmental exposures with indoor sources due to new-fashioned lifestyles that have developed as the economy has grown.

## Supporting Information

S1 Table Different indicators of rapid modernization in Shanghai from 1990 to 2011. (DOCX)

S2 Table PRISMA (Preferred Reporting Items for Systematic Reviews and Meta-Analyses) checklist for the systematic review. (DOCX)

S3 Table Questions for the studied diseases in our questionnaire for the present study. (DOCX)

S4 Table Detailed sample numbers for different age groups as provided in the selected studies. (DOCX)

S5 Table Detailed sample numbers among different districts of Shanghai as provided in the selected studies. (DOCX)

S6 Table Detailed data for prevalences of wheeze, rhinitis, and eczema in different ages during lifetime since birth (ever). (DOCX)

S7 Table Recalculated prevalences of asthma in different age groups (infancy, preschool age, school age, puberty (DOCX)

S8 Table Concentrations of outdoor air pollutants in Shanghai from 2001 to 2011. (DOCX)

S1 Fig Distribution of the percentage of children for total episodes of common cold in the last 12 months. (DOCX)

S2 Fig Time-trend prevalences of childhood asthma in different districts. Herein the data in red frame are the prevalences in the different urban districts (A~I).
(DOCX)
