# Supplementary material for: Updated Prevalences of Asthma, Allergy, and Airway Symptoms, and a Systematic Review of Trends over Time for Childhood Asthma in Shanghai, China
Source: PLoS One. 2015 Apr 13;10(4):e0121577. doi: 10.1371/journal.pone.0121577 (PMC4395352; doi:10.1371/journal.pone.0121577)
Supplement: S1 Table — (DOCX) [file pone.0121577.s001.docx]

**S1 Table.** Different indicators of rapid modernization in Shanghai from 1990 to 2011^a^.

| Year | PDI of urban residents  (yuan in CNY) | PDI of rural residents  (yuan in CNY) | Annual energy consumption  (ten thousand tons standard coal) | Total emissions of  waste gases  (hundred millions m^3^) | Resident population  (ten thousands) |
| --- | --- | --- | --- | --- | --- |
| 1990 | 2183 | 1665 | 3191.06 | NA | 1334.00 |
| 1991 | 2486 | NA | NA | NA | 1350.00 |
| 1992 | 3009 | NA | NA | NA | 1350.00 |
| 1993 | 4277 | NA | NA | NA | 1365.00 |
| 1994 | 5868 | NA | NA | NA | 1381.00 |
| 1995 | 7172 | 4246 | 4465.87 | 4625 | 1398.00 |
| 1996 | 8159 | NA | 4626.21 | 4757 | 1414.00 |
| 1997 | 8439 | NA | 4758.82 | 4755 | 1451.00 |
| 1998 | 8773 | NA | 4874.11 | 4912 | 1489.00 |
| 1999 | 10932 | NA | 5119.19 | 4947 | 1527.00 |
| 2000 | 11718 | 5565 | 5499.48 | 5755 | 1567.00 |
| 2001 | 12883 | 5850 | 5894.78 | 6964 | 1608.00 |
| 2002 | 13250 | 6212 | 6249.34 | 7440 | 1668.33 |
| 2003 | 14867 | 6658 | 6796.34 | 7799 | 1712.97 |
| 2004 | 16683 | 7337 | 7405.64 | 8834 | 1765.84 |
| 2005 | 18645 | 8342 | 8225.05 | 8482 | 1890.26 |
| 2006 | 20668 | 9213 | 8875.70 | 9428 | 1964.11 |
| 2007 | 23623 | 10222 | 9670.45 | 9591 | 2063.58 |
| 2008 | 26675 | 11385 | 10207.36 | 10436 | 2140.65 |
| 2009 | 28838 | 12324 | 10367.38 | 10059 | 2210.28 |
| 2010 | 31838 | 13746 | 11201.13 | 12969 | 2302.66 |
| 2011 | 36230 | 15644 | 11270.48 | 13692 | 2347.46 |

^a^ PDI: Per-capita Disposable Income; NA: not available. All data derived from Shanghai Statistical Yearbook 2012 [1].

Reference

1. Shanghai Municipal Bureau of Statistics (2012) Shanghai Statistical Yearbook 2012 (In Chinese). China Statistics Press.
