# Supplementary material for: Updated Prevalences of Asthma, Allergy, and Airway Symptoms, and a Systematic Review of Trends over Time for Childhood Asthma in Shanghai, China
Source: PLoS One. 2015 Apr 13;10(4):e0121577. doi: 10.1371/journal.pone.0121577 (PMC4395352; doi:10.1371/journal.pone.0121577)
Supplement: S3 Table — (DOCX) [file pone.0121577.s003.docx]

**S3 Table.** Questions for the studied diseases in our questionnaire for the present study.

| Disease | Question | Answer |
| --- | --- | --- |
| Wheeze, ever | Has your child ever had wheezing or whistling in the chest at any time in the past? (More than one alternative possible) | - Yes, prior to 1 year of age - Yes, 1-2 years of age - Yes, 3-4 years of age - Yes, more than 4 years of age - No |
| Wheeze in last 12 months | Has your child had wheezing or whistling in the chest in the last 12 months? (More than one alternative possible) | - Yes, when having a cold - Yes, during exercise - Yes, when playing or being outdoors - Yes, when laughing or crying - Yes, in contact with furred animals - No |
| Dry cough in last 12 months | In the last 12 months, has your child had a dry cough at night for more than two weeks, apart from a cough associated with a cold or chest infection? | - Yes - No |
| Doctor diagnosed asthma | Has your child been diagnosed with asthma by a doctor? | - Yes - No |
| Croup, ever | Has your child had croup (breathing difficulties with server (dry) cough)? | - Yes - No |
| Pneumonia, ever | Has your child been diagnosed with pneumonia by a doctor? | - Yes - No |
| Rhinitis, ever | Has your child ever had a problem with sneezing, or a runny, or a blocked nose when he / she did not have a cold or a flu? (More than one alternative possible) | - Yes, prior to 1 year of age - Yes, 1-2 years of age - Yes, 3-4 years of age - Yes, more than 4 years of age - No |
| Rhinitis in last 12 months | In the past 12 months, has your child had a problem with sneezing, or a runny, or a blocked nose when he / she did not have a cold or the flu? | - Yes - No |
| Rhinitis on pet exposure | In the past 12 months, has your child had a problem with sneezing, a runny or a blocked nose, or itchy-watery eyes after been in contact with furred animals? | - Yes - No |
| Rhinitis on pollen exposure | In the past 12 months, has your child had a problem with sneezing, a runny or a blocked nose, or itchy-watery eyes after been in contact with pollen? | - Yes - No |
| Doctor diagnosed hay fever | Has your child been diagnosed with hay fever or allergic rhinitis by a doctor? | - Yes - No |

**S3 Table.** Continued.

| Eczema, ever | Has your child ever had an itchy rash (eczema), which was coming and going for the last 6 months? | - Yes, prior to 1 year of age - Yes, 1-2 years of age - Yes, 3-4 years of age - Yes, after 4 years of age - No |
| --- | --- | --- |
| Eczema in last 12 months | Has your child had this itchy rash at any time in the last 12 months? | - Yes - No |
| Food allergy | Has the child at any time had allergic irritations such as eczema, nettle-rash, diarrhoea, swollen lips or eyes caused by the listed foods below? (More than one alternative possible) | - Yes, egg - Yes, sea food - Yes, meat - Yes, vegetable - Yes, flour - Yes, bean - Yes, fruit - Yes, milk or dairy product - Yes, nut, (peanut, walnut etc.) - Yes, other - No - Not known |
| Otitis media | Has your child ever had inflammations of the ears? | - Yes, 1 – 2 times - Yes, 3 – 5 times - Yes, more than 5 times - No |
| Common cold | In the past 12 months, how many times has your child had a cold? | - Less than 3 times - 3– 5 times - 6 – 10 times - More than 10 times |
