# Supplementary material for: Updated Prevalences of Asthma, Allergy, and Airway Symptoms, and a Systematic Review of Trends over Time for Childhood Asthma in Shanghai, China
Source: PLoS One. 2015 Apr 13;10(4):e0121577. doi: 10.1371/journal.pone.0121577 (PMC4395352; doi:10.1371/journal.pone.0121577)
Supplement: S4 Table — (DOCX) [file pone.0121577.s004.docx]

**S4 Table.** Detailed sample numbers for different age groups as provided in the selected studies.

|  | | Year | | | | | | | | | | | | | | | | | | | | | | | | | | | | | | | | |
| --- | --- | --- | --- | --- | --- | --- | --- | --- | --- | --- | --- | --- | --- | --- | --- | --- | --- | --- | --- | --- | --- | --- | --- | --- | --- | --- | --- | --- | --- | --- | --- | --- | --- | --- |
| Age | | 1990 [1] | | | | | | | | | | | | | | | | | |  | 2000 [2] | | | | | | | | | | | | | |
|  |  | Male | | | | |  | Female | | | | |  | | Total | | | | |  | Male | | | | |  | Female | | | |  | Total | | |
|  |  | *N* | | *n* (P,%) ^a)^ | | |  | *N* | | *n* (P,%) ^a)^ | | |  | | *N* | *n* (P,%) ^a)^ | | | |  | *N* | | *n* (P,%) ^a)^ | | |  | *N* | | *n* (P,%) ^a)^ | |  | *N* | | *n* (P,%) ^a)^ |
| 0 | | 866 | | 8(0.92) | | |  | 766 | | 2(2.61) | | |  | | 1632 | 10(0.61) | | | |  | 320 | | 0(0) | | |  | 291 | | 0(0) | |  | 611 | | 0(0) |
| 1 | | 1260 | | 29(2.30) | | |  | 1129 | | 11(0.97) | | |  | | 2389 | 40(1.67) | | | |  | 316 | | 3(0.95) | | |  | 300 | | 2(0.67) | |  | 616 | | 5(0.81) |
| 2 | | 1566 | | 50(3.19) | | |  | 1458 | | 22(1.50) | | |  | | 3024 | 72(2.38) | | | |  | 368 | | 18(4.89) | | |  | 345 | | 7(2.03) | |  | 713 | | 25(3.51) |
| 3 | | 1680 | | 58(3.45) | | |  | 1581 | | 24(1.51) | | |  | | 3261 | 82(2.51) | | | |  | 438 | | 29(6.62) | | |  | 426 | | 11(2.58) | |  | 864 | | 40(4.63) |
| 4 | | 1700 | | 44(2.58) | | |  | 1483 | | 13(0.87) | | |  | | 3183 | 57(1.79) | | | |  | 456 | | 31(6.80) | | |  | 431 | | 16(3.71) | |  | 887 | | 47(5.30) |
| 5 | | 1717 | | 38(2.21) | | |  | 1499 | | 22(1.46) | | |  | | 3216 | 60(1.87) | | | |  | 444 | | 28(6.31) | | |  | 422 | | 10(2.37) | |  | 866 | | 38(4.39) |
| 6 | | 1756 | | 45(2.56) | | |  | 1645 | | 24(1.45) | | |  | | 3401 | 69(2.03) | | | |  | 458 | | 27(5.90) | | |  | 453 | | 20(4.42) | |  | 911 | | 47(5.16) |
| 7 | | 1882 | | 63(3.35) | | |  | 1800 | | 23(1.28) | | |  | | 3682 | 86(2.34) | | | |  | 420 | | 28(6.67) | | |  | 425 | | 12(2.82) | |  | 845 | | 40(4.73) |
| 8 | | 1543 | | 31(2.00) | | |  | 1579 | | 26(1.64) | | |  | | 3127 | 57(1.82) | | | |  | 436 | | 27(6.19) | | |  | 467 | | 15(3.21) | |  | 903 | | 42(4.65) |
| 9 | | 1238 | | 30(2.42) | | |  | 1197 | | 20(1.67) | | |  | | 2435 | 50(2.05) | | | |  | 533 | | 45(8.44) | | |  | 458 | | 18(3.93) | |  | 991 | | 63(6.36) |
| 10 | | 1015 | | 18(1.77) | | |  | 965 | | 9(0.93) | | |  | | 1930 | 27(1.36) | | | |  | 640 | | 51(7.97) | | |  | 567 | | 27(4.76) | |  | 1207 | | 78(6.46) |
| 11 | | 842 | | 15(1.78) | | |  | 825 | | 4(0.48) | | |  | | 1667 | 19(1.14) | | | |  | 624 | | 50(8.01) | | |  | 647 | | 28(4.33) | |  | 1271 | | 78(6.14) |
| 12 | | 733 | | 15(2.05) | | |  | 687 | | 6(0.87) | | |  | | 1420 | 21(1.48) | | | |  | 711 | | 50(7.03) | | |  | 655 | | 20(3.05) | |  | 1367 | | 70(5.12) |
| 13 | | 909 | | 17(1.87) | | |  | 811 | | 1(0.12) | | |  | | 1720 | 18(1.05) | | | |  | 630 | | 32(5.08) | | |  | 668 | | 20(2.99) | |  | 1298 | | 52(4.01) |
| 14 | | 1125 | | 7(0.62) | | |  | 1026 | | 11(1.07) | | |  | | 2151 | 18(0.84) | | | |  | 562 | | 20(3.56) | | |  | 551 | | 9(1.63) | |  | 1113 | | 29(2.61) |
| Total | | 19837 | | 468 | | |  | 18451 | | 218 | | |  | | 38288 | 686(1.79) | | | |  | 7356 | | 439(5.97) | | |  | 7107 | | 215(3.03) | |  | 14462 | | 654(4.52) |
|  | Year | | | | | | | | | | | | | | | | | | | | | | | | | | | | | | | | | |
| Age | 2006 [3] | | | | | | | | | | | | | | | |  | 2007 [4] | | | |  | | 2008 [5] ^b)^ | | | | | | | | | | |
|  | Male | | | |  | Female | | | | |  | Total | | | | |  | Total | | | |  | | Male | | | |  | Female | | |  | Total | |
|  | *N* | | *n* (P,%) ^a)^ | |  | *N* | | | *n* (P,%) ^a)^ | |  | *N* | | *n* (P,%) ^a)^ | | |  | *N* | *n* (P,%) ^a)^ | | |  | | *N* | *n* (P,%) ^a)^ | | |  | *N* | *n* (P,%) ^a)^ | |  | *N* | *n* (P,%) ^a)^ |
| 1 |  | |  | |  |  | | |  | |  |  | |  | | |  |  |  | | |  | | 320 | 36(11.25) | | |  | 306 | 27(8.82) | |  | 625 | 63(10.06) |
| 2 |  | |  | |  |  | | |  | |  |  | |  | | |  |  |  | | |  | | 155 | 17(10.90) | | |  | 115 | 10(8.70) | |  | 270 | 27(10.00) |
| 3 |  | |  | |  |  | | |  | |  |  | |  | | |  |  |  | | |  | | 346 | 35(10.12) | | |  | 357 | 30(8.40) | |  | 703 | 65(9.25) |
| 4 |  | |  | |  |  | | |  | |  |  | |  | | |  | 145 | 12(8.3) | | |  | | 62 | 15(24.19) | | |  | 49 | 4(8.16) | |  | 111 | 19(17.12) |
| 5 |  | |  | |  |  | | |  | |  |  | |  | | |  | 161 | 12(7.5) | | |  | | 198 | 24(12.12) | | |  | 203 | 15(7.34) | |  | 401 | 39(9.73) |
| 6 | 266 | | 18(6.77) | |  | 233 | | | 10(4.29) | |  | 499 | | 28(5.61) | | |  | 169 | 13(7.7) | | |  | | 218 | 31(14.22) | | |  | 173 | 21(12.14) | |  | 391 | 52(13.40) |
| 7 | 396 | | 35(8.84) | |  | 350 | | | 30(8.57) | |  | 746 | | 65(8.71) | | |  | 606 | 36(5.9) | | |  | | 330 | 41(12.42) | | |  | 321 | 37(11.56) | |  | 651 | 78(11.98) |
| 8 | 411 | | 39(9.49) | |  | 359 | | | 12(3.34) | |  | 770 | | 51(6.62) | | |  | 737 | 35(4.7) | | |  | | 249 | 33(13.25) | | |  | 237 | 11(4.64) | |  | 486 | 44(9.05) |
| 9 | 432 | | 40(9.26) | |  | 362 | | | 23(6.35) | |  | 794 | | 63(7.93) | | |  | 732 | 27(3.7) | | |  | | 242 | 26(10.74) | | |  | 257 | 20(7.78) | |  | 499 | 46(9.22) |
| 10 | 389 | | 32(8.23) | |  | 347 | | | 15(4.32) | |  | 736 | | 47(6.39) | | |  | 685 | 34(5.0) | | |  | | 248 | 32(12.90) | | |  | 213 | 16(7.51) | |  | 461 | 48(10.41) |
| 11 | 441 | | 37(8.39) | |  | 434 | | | 17(3.92) | |  | 875 | | 54(6.17) | | |  | 578 | 23(4.0) | | |  | | 246 | 28(11.38) | | |  | 250 | 17(6.08) | |  | 496 | 45(9.07) |
| 12 | 504 | | 34(6.75) | |  | 537 | | | 14(2.61) | |  | 1041 | | 48(4.61) | | |  | 288 | 8(2.8) | | |  | | 229 | 22(9.61) | | |  | 235 | 9(3.83) | |  | 464 | 31(6.68) |
| 13 | 517 | | 26(5.03) | |  | 533 | | | 18(3.38) | |  | 1050 | | 44(4.19) | | |  | 573 | 28(4.9) | | |  | | 277 | 19(6.86) | | |  | 275 | 6(2.18) | |  | 552 | 25(4.53) |
| 14 | 319 | | 12(3.76) | |  | 296 | | | 10(3.38) | |  | 615 | | 22(3.58) | | |  | 847 | 33(3.9) | | |  | | 412 | 32(7.77) | | |  | 398 | 30(7.54) | |  | 810 | 62(7.65) |
| Total | 3675 | | 273(7.43) | |  | 3451 | | | 149(4.32) | |  | 7126 | | 422(5.92) | | |  | 5521 | 226(4.09) | | |  | | 3532 | 391(11.07) | | |  | 3389 | 244(7.20) | |  | 6920 | 635(9.18) |

^a)^ “*N*” is the total number of children in different groups; “*n*” is the number of children who had asthma; “P” is prevalence.

^b)^ Prevalences for 2008 include wheezing as well as asthma ever.

Reference

1. The Cooperation Group on Childhood Asthma of Shanghai Medical Association (1994) The report of a cluster sampling survey for asthmatic symptoms among 0-14 years old children in Shanghai (in Chinese). Journal of Clinical Pediatrics 12 (2): 107-109.
2. The Cooperation Group on Childhood Asthma of Shanghai Medical Association (2002) the survey for bronchial asthma among 0-14 years old children in Shanghai (in Chinese). Journal of Clinical Pediatrics 12(2): 144-147.
3. Yuan D, Shen CL, Jiang ZH, Huang HT, Gao HM, et al. (2007) An investigation on the prevalence of asthma and its influence factors among school age children in shanghai (in Chinese). Journal of Environmental & Occupational Medicine 24 (6): 573-576.
4. Niu CJ, Wu JG, Zhuang ZJ, Ru GL, Tang CX (2010) Prevalence of respiratory symptoms and diseases among children and adolescent in urban Shanghai (in Chinese). Chinese Journal of School Health 31(6): 708-710.
5. Zhang YE, Shan BL, Yu J, Chen J, Wei L, et al. (2012) The epidemiological survey of children’s wheezing and asthma in Putuo district in Shanghai (in Chinese). Journal of Clinical Pediatrics 30(4): 339-341.
