# Supplementary material for: Updated Prevalences of Asthma, Allergy, and Airway Symptoms, and a Systematic Review of Trends over Time for Childhood Asthma in Shanghai, China
Source: PLoS One. 2015 Apr 13;10(4):e0121577. doi: 10.1371/journal.pone.0121577 (PMC4395352; doi:10.1371/journal.pone.0121577)
Supplement: S5 Table — (DOCX) [file pone.0121577.s005.docx]

**S5 Table.** Detailed sample numbers among different districts of Shanghai as provided in the selected studies.

|  | Year | | | | | | | | | | |
| --- | --- | --- | --- | --- | --- | --- | --- | --- | --- | --- | --- |
|  | 1990 [1] | | |  | 2000 [2] | | |  | 2006 [3] | | |
|  | *N* | *n* | P (%) ^a)^ |  | *N* | *n* | P (%) ^a)^ |  | *N* | *n* | P (%) ^a)^ |
| **District** |  |  |  |  |  |  |  |  |  |  |  |
| Hong-Kou | 4329 | 57 | 1.317 |  | 3632 | 149 | 4.1 |  |  |  |  |
| Bao-Shan | 4007 | 48 | 1.198 |  | 1500 | 61 | 4.07 |  |  |  |  |
| Jing-An | 4036 | 83 | 2.056 |  |  |  |  |  |  |  |  |
| Xu-Hui | 5248 | 95 | 1.81 |  | 2951 | 128 | 4.34 |  |  |  |  |
| Zha-Bei | 4346 | 40 | 0.92 |  |  |  |  |  | 1629 | 125 | 7.67 |
| Huang-Pu | 4228 | 121 | 2.861 |  | 2752 | 120 | 4.36 |  |  |  |  |
| Ru-Wan | 4537 | 102 | 2.248 |  | 2500 | 138 | 5.52 |  | 1911 | 165 | 8.63 |
| Chang-Ni | 2181 | 51 | 2.338 |  |  |  |  |  |  |  |  |
| Yang-Pu | 5376 | 89 | 1.656 |  |  |  |  |  | 1683 | 105 | 6.24 |
| Pu-Tuo |  |  |  |  | 1127 | 58 | 5.15 |  |  |  |  |
| Qing-Pu |  |  |  |  |  |  |  |  | 1903 | 27 | 1.42 |
| **Total** | 38288 | 686 | 1.791 |  | 14462 | 654 | 4.52 |  |  |  |  |

^a)^ “*N*” is the total number of children in different groups; “*n*” is the number of children who had asthma; “P” is prevalence.

Reference

1. The Cooperation Group on Childhood Asthma of Shanghai Medical Association (1994) The report of a cluster sampling survey for asthmatic symptoms among 0-14 years old children in Shanghai (in Chinese). Journal of Clinical Pediatrics 12 (2): 107-109.
2. The Cooperation Group on Childhood Asthma of Shanghai Medical Association (2002) the survey for bronchial asthma among 0-14 years old children in Shanghai (in Chinese). Journal of Clinical Pediatrics 12(2): 144-147.
3. Yuan D, Shen CL, Jiang ZH, Huang HT, Gao HM, et al. (2007) An investigation on the prevalence of asthma and its influence factors among school age children in shanghai (in Chinese). Journal of Environmental & Occupational Medicine 24 (6): 573-576.
