# Supplementary material for: Updated Prevalences of Asthma, Allergy, and Airway Symptoms, and a Systematic Review of Trends over Time for Childhood Asthma in Shanghai, China
Source: PLoS One. 2015 Apr 13;10(4):e0121577. doi: 10.1371/journal.pone.0121577 (PMC4395352; doi:10.1371/journal.pone.0121577)
Supplement: S6 Table — (DOCX) [file pone.0121577.s006.docx]

**S6 Table.** Detailed data for prevalences of wheeze, rhinitis, and eczema in different ages during lifetime since birth (ever).

|  | Prevalence, *N* (%) | | | | | | | | | | | | | | | | | | |
| --- | --- | --- | --- | --- | --- | --- | --- | --- | --- | --- | --- | --- | --- | --- | --- | --- | --- | --- | --- |
|  | Before 1 year old | | | |  | 1-2 years old | | | |  | 3-4 years old | | | |  | After 4 years old | | | |
|  | Total | Male | Female | *p* |  | Total | Male | Female | *p* |  | Total | Male | Female | *p* |  | Total | Male | Female | *p* |
| Wheeze | 546  (3.7) | 357  (4.8) | 187  (2.6) | <0.001 |  | 1233  (8.5) | 765  (10.4) | 464  (6.5) | <0.001 |  | 1928  (13.2) | 1038  (14.1) | 888  (12.4) | 0.003 |  | 706  (4.8) | 343  (4.7) | 361  (5.1) | 0.261 |
| Rhinitis | 1373  (9.5) | 737  (10.1) | 632  (8.9) | 0.016 |  | 2764  (19.2) | 1475  (20.2) | 1278  (18.0) | 0.001 |  | 3870  (26.7) | 2031  (27.8) | 1828  (25.7) | 0.006 |  | 1978  (13.7) | 1021  (14.0) | 950  (13.4) | 0.301 |
| Eczema | 1040  (7.6) | 572  (8.2) | 460  (6.8) | 0.001 |  | 1046  (7.6) | 512  (7.4) | 530  (7.8) | 0.308 |  | 769  (5.6) | 389  (5.6) | 373  (5.5) | 0.823 |  | 351  (2.6) | 168  (2.4) | 180  (2.7) | 0.369 |
