# Supplementary material for: Updated Prevalences of Asthma, Allergy, and Airway Symptoms, and a Systematic Review of Trends over Time for Childhood Asthma in Shanghai, China
Source: PLoS One. 2015 Apr 13;10(4):e0121577. doi: 10.1371/journal.pone.0121577 (PMC4395352; doi:10.1371/journal.pone.0121577)
Supplement: S8 Table — (DOCX) [file pone.0121577.s008.docx]

**S8 Table.** Concentrations of outdoor air pollutants in Shanghai from 2001 to 2011.

| Year | Concentration (mg/m^3^) | | | Reference |
| --- | --- | --- | --- | --- |
|  | Sulfur dioxide (SO_2_) | Inhalable particles (PM10) | Nitrogen dioxide (NO_2_) |  |
| 2001 | 0.063 | 0.100 | 0.043 | [1] |
| 2002 | 0.035 | 0.108 | 0.058 | [2] |
| 2003 | 0.043 | 0.097 | 0.057 | [3] |
| 2004 | 0.055 | 0.099 | 0.062 | [4] |
| 2005 | 0.061 | 0.088 | 0.061 | [5] |
| 2006 | 0.051 | 0.086 | 0.055 | [6] |
| 2007 | 0.055 | 0.088 | 0.054 | [7] |
| 2008 | 0.051 | 0.084 | 0.056 | [8] |
| 2009 | 0.035 | 0.081 | 0.053 | [9] |
| 2010 | 0.029 | 0.079 | 0.050 | [10] |
| 2011 | 0.029 | 0.080 | 0.051 | [11] |

Reference

1. Hong H (2002) Shanghai Environment Yearbook 2002 (in Chinese). Shanghai People Publishing House.
2. Xu ZX (2003) Shanghai Environment Yearbook 2003 (in Chinese). Shanghai People Publishing House.
3. Xu ZX (2004) Shanghai Environment Yearbook 2004 (in Chinese). Shanghai People Publishing House.
4. Xu ZX (2005) Shanghai Environment Yearbook 2005 (in Chinese). Shanghai People Publishing House.
5. Xu ZX (2006) Shanghai Environment Yearbook 2006 (in Chinese). Shanghai People Publishing House.
6. Zhang Q (2007) Shanghai Environment Yearbook 2007 (in Chinese). Shanghai People Publishing House.
7. Zhang Q (2008) Shanghai Environment Yearbook 2008 (in Chinese). Shanghai People Publishing House.
8. Zhang Q (2009) Shanghai Environment Yearbook 2009 (in Chinese). Shanghai People Publishing House.
9. Zhang Q (2010) Shanghai Environment Yearbook 2010 (in Chinese). Shanghai People Publishing House.
10. Zhang Q (2011) Shanghai Environment Yearbook 2011 (in Chinese). Shanghai People Publishing House.
11. Zhang Q (2012) Shanghai Environment Yearbook 2012 (in Chinese). Shanghai People Publishing House.
