# Supplementary material for: Updated Prevalences of Asthma, Allergy, and Airway Symptoms, and a Systematic Review of Trends over Time for Childhood Asthma in Shanghai, China
Source: PLoS One. 2015 Apr 13;10(4):e0121577. doi: 10.1371/journal.pone.0121577 (PMC4395352; doi:10.1371/journal.pone.0121577)
Supplement: S1 Fig — (DOCX) [file pone.0121577.s009.docx]

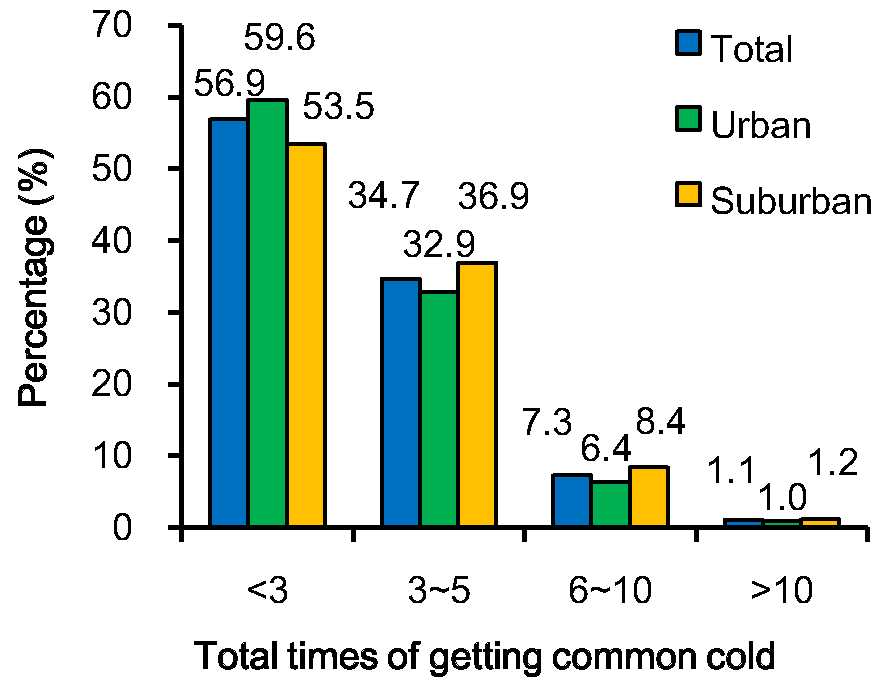


**S1 Fig**. Distribution of the percentage of children for total episodes of common cold in the last 12 months.
