# Supplementary material for: Updated Prevalences of Asthma, Allergy, and Airway Symptoms, and a Systematic Review of Trends over Time for Childhood Asthma in Shanghai, China
Source: PLoS One. 2015 Apr 13;10(4):e0121577. doi: 10.1371/journal.pone.0121577 (PMC4395352; doi:10.1371/journal.pone.0121577)
Supplement: S2 Fig — Herein the data in red frame are the prevalences in the different urban districts (A~I). (DOCX) [file pone.0121577.s010.docx]

**
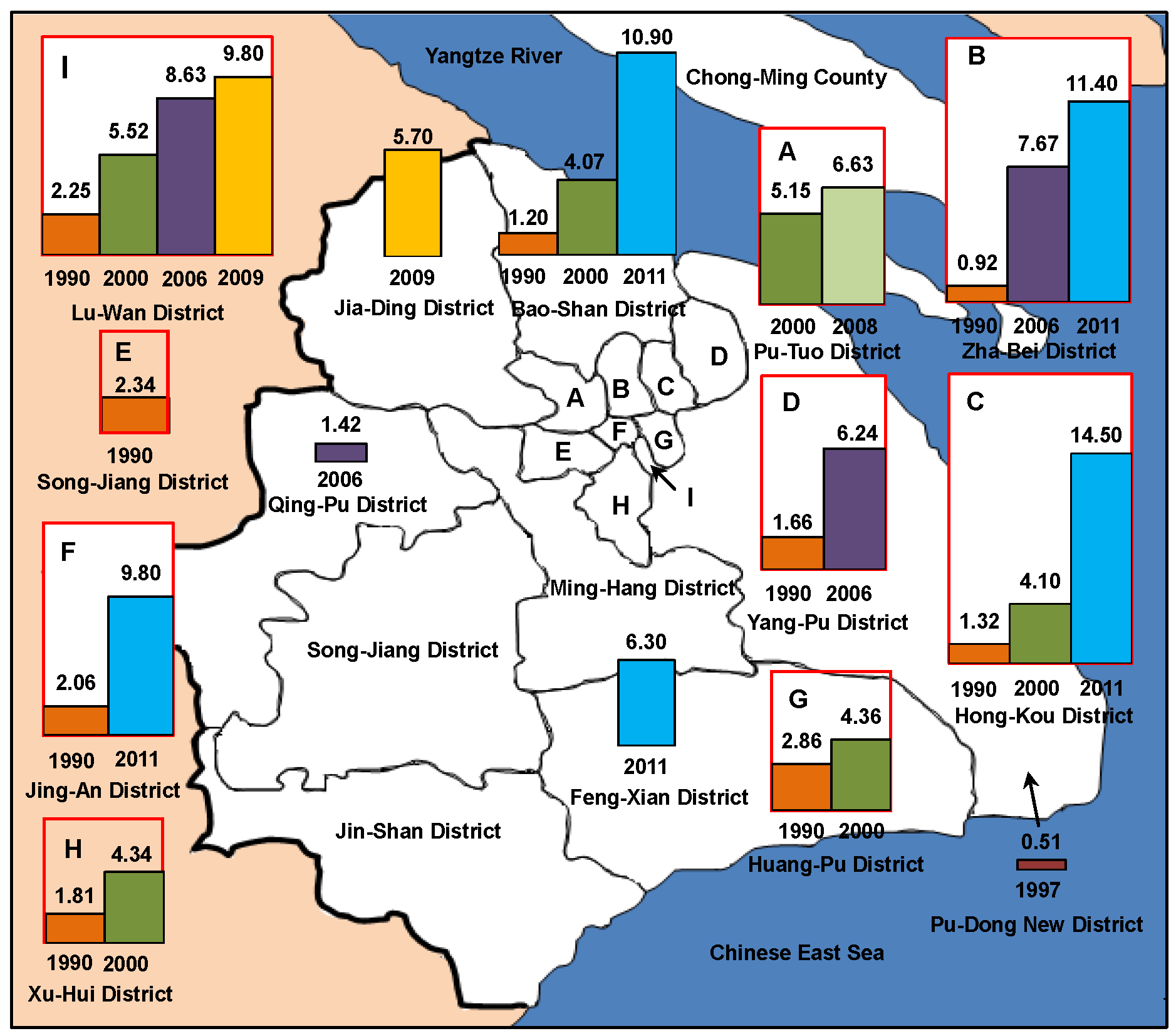
 S2 Fig.** Time-trend prevalences of childhood asthma in different districts. Herein the data in red frame are the prevalences in the different urban districts (A~I). References: 1990 [1]; 1997 [2]; 2000 [3]; 2006 [4]; 2008 [5]; 2009 [6].

Reference

1. The Cooperation Group on Childhood Asthma of Shanghai Medical Association (1994) The report of a cluster sampling survey for asthmatic symptoms among 0-14 years old children in Shanghai (in Chinese). Journal of Clinical Pediatrics 12 (2): 107-109.
2. Hang JQ, Sun BX, Dai HL, Guo HY, Zhou CP, et al. (2002) The epidemiological survey of bronchial asthma among 40 thousands population in the Jinqiao area of Pudong district, Shanghai (in Chinese). Chinese Journal of Practical Internal Medicine 22(10): 616-617.
3. The Cooperation Group on Childhood Asthma of Shanghai Medical Association (2002) the survey for bronchial asthma among 0-14 years old children in Shanghai (in Chinese). Journal of Clinical Pediatrics 12(2): 144-147.
4. Yuan D, Shen CL, Jiang ZH, Huang HT, Gao HM, et al. (2007) An investigation on the prevalence of asthma and its influence factors among school age children in shanghai (in Chinese). Journal of Environmental & Occupational Medicine 24 (6): 573-576.
5. Zhang YE, Shan BL, Yu J, Chen J, Wei L, et al. (2012) The epidemiological survey of children’s wheezing and asthma in Putuo district in Shanghai (in Chinese). Journal of Clinical Pediatrics 30(4): 339-341.
6. Dong W, Zhou Y, Su W, Sheng J, Li YZ, et al. (2012) Relationship of multiple risk factors in infancy with asthma morbidity at age 7 to 8 years in suburb and urban of Shanghai (in Chinese). Journal of Clinical Pediatrics 30(12):1139-1143.
